# Plasma 24S-hydroxycholesterol levels in elderly subjects with late onset Alzheimer's disease or vascular dementia: a case-control study

**DOI:** 10.1186/1471-2377-11-121

**Published:** 2011-10-04

**Authors:** Giovanni Zuliani, Michela Perrone Donnorso, Cristina Bosi, Angelina Passaro, Edoardo Dalla Nora, Amedeo Zurlo, Francesco Bonetti, Alessia F Mozzi, Claudio Cortese

**Affiliations:** 1 Department of Clinical and Experimental Medicine, Section of Internal Medicine, Gerontology and Clinical Nutrition; Azienda Ospedaliero-Universitaria Arcispedale S. Anna, Ferrara, Italy; 2 Associazione Alzheimer-Perusini, Ferrara, Italy; 3 Department of Clinical Biochemistry and Molecular Biology, University of Rome 2, Tor Vergata, Italy; 4 Geriatrics Division; Azienda Ospedaliero-Universitaria Arcispedale S. Anna, Ferrara, Italy

## Abstract

**Background:**

In central nervous system cholesterol cannot be degraded but is secreted into circulation predominantly in the form of its polar metabolite 24(*S*)-hydroxycholesterol (24S-OH-Chol). Some studies suggested an association between 24S-OH-Chol metabolism and different neurological diseases including dementia. A possible decrease in 24S-OH-Chol plasma levels has been reported late onset Alzheimer's disease (LOAD) and vascular dementia (VD), but results of previous studies are partially contradictory.

**Methods:**

By high-speed liquid chromatography/tandem mass spectrometry we evaluated the plasma levels of 24S-OH-Chol in a sample of 160 older individuals: 60 patients with LOAD, 35 patients with VD, 25 subjects affected by cognitive impairment no-dementia (CIND), and 40 (144 for genetics study) cognitively normal Controls. We also investigated the possible association between PPARgamma Pro12Ala polymorphism and dementia or 24S-OH-Chol levels.

**Results:**

Compared with Controls, plasma 24S-OH-Chol levels were higher in LOAD and lower in VD; a slight not-significant increase in CIND was observed (ANOVA p: 0.001). A positive correlation between 24S-OH-Chol/TC ratio and plasma C reactive protein (CRP) levels was found in the whole sample, independent of possible confounders (multiple regression p: 0.04; r^2^: 0.10). This correlation was strong in LOAD (r: 0.39), still present in CIND (r: 0.20), but was absent in VD patients (r: 0.08). The PPARgamma Pro12Ala polymorphism was not associated with the diagnosis of LOAD, VD, or CIND; no correlation emerged between the Ala allele and 24S-OH-Chol plasma levels.

**Conclusions:**

Our results suggest that plasma 24S-OH-Chol levels might be increased in the first stages of LOAD, and this phenomenon might be related with systemic inflammation. The finding of lower 24S-OH-Chol concentrations in VD might be related with a more advanced stage of VD compared with LOAD in our sample, and/or to different pathogenetic mechanisms and evolution of these two forms of dementia.

## Background

Late onset Alzheimer's disease (LOAD) is the most common form of dementia in older individuals living in Western countries, followed by vascular dementia (VD) [1,2]. Besides a definite role in the pathogenesis of atherosclerosis, and of consequence of some types of VD, disturbances of cholesterol homeostasis might have a role also in the development of LOAD [3,4]. In central nervous system (CNS) cholesterol originates almost exclusively from *in situ *synthesis [5], while circulating cholesterol is normally prevented from entering the CNS by the blood-brain-barrier [4]. As cholesterol cannot be eliminated in CNS, and may be toxic to neurons when in excess, it is secreted from CNS into the circulation predominantly in the form of its polar metabolite 24(*S*)-hydroxycholesterol (24S-OH-Chol) [6].

Interestingly, some studies have suggested a possible association between 24S-OH-Chol metabolism and different neurological diseases [7]. As most of the circulating 24S-OH-Chol originates in the brain, it has been proposed that the concentrations of 24S-OH-Chol in cerebrospinal fluid and/or plasma might represent peripheral markers of neuronal degeneration occurring in primary diseases of CNS [8].

It has been reported that 24S-OH-Chol levels were higher in cerebrospinal fluid from LOAD patients compared to controls [9,10]. On the contrary, 24S-OH-Chol concentrations were found to be low in brain samples obtained from deceased patients with LOAD in advanced stage [11].

Plasma levels of 24S-OH-Chol should reflect the number of active neurons in the brain and thus, the volume of the grey matter structures. In different neurodegenerative disorders plasma 24S-OH-Chol was found to be reduced, proportionally to the degree of brain atrophy as measured by MRI [8]. As regards dementia, only a few studies conducted on small samples of patients have investigated this specific topic. At first, slightly higher 24S-OH-Chol levels have been found, both in patients with LOAD and VD [12]. Subsequent studies gave different results reporting normal [10] or even decreased 24S-OH-Chol levels in patients with LOAD or VD compared with controls [13-15]. Of consequence, findings about 24S-OH-Chol plasma levels in patients affected by LOAD and VD are not definite; indeed, it is possible that brain cholesterol and thus 24S-OH-Chol plasma levels might change over time along these two neurological diseases.

Some studies have also investigated the possible influence of common DNA polymorphisms on 24S-OH-Chol plasma levels or the risk of being affected by LOAD. In particular, the genes coding for the 24S-hydroxylase (CYP46) and for the peroxisome proliferator-activated receptor gamma (PPARgamma) have been studied [16,17].

In the present study we evaluated, by high-speed liquid chromatography/tandem mass spectrometry, the plasma levels of 24S-OH-Chol in a sample of older individuals affected by LOAD, VD, or cognitive impairment no dementia (CIND); successively, we investigated the possible association between the PPARgamma Pro12Ala polymorphism and 24S-OH-Chol levels.

## Methods

### Subjects

On the whole, 120 consecutive patients referring to the Day Hospital service for the study of cognitive decline (Institute of Internal Medicine, Gerontology and Clinical Nutrition; Geriatric Unit, S. Anna University-Hospital, Ferrara, Italy) were enrolled. All data were collected during a three years period (2006-2009). The sample included:

1) Sixty patients with LOAD (mean age: 78 ± 5.5 SD years; females: 78%) by the NINCDS-ADRDA criteria [18]. Only patients with "probable" LOAD were included; patients with "possible" LOAD or with LOAD associated with significant cerebrovascular disease on CT scan were excluded in order to increase specificity. The Global Deterioration Scale ranged from stage 3 to 5.

2) Thirty five patients with VD (mean age: 77 ± 7.6 SD years; females: 60%) by the NINDS-AIREN criteria [19]. Only patients with "probable" VD were enrolled. The Global Deterioration Scale ranged from stage 4 to 6.

3) Twenty five patients with cognitive impairment no dementia (CIND) (mean age: 74 ± 8.2 SD years; females: 44.5%). CIND was defined as the presence of short/long-term memory impairment and/or impairment in other single or multiple cognitive domains in an individual who didn't meet the standardized criteria for dementia. We also required that the patient with CIND would be still independent in the activities of daily living (ADLs). Most of these individuals were affected by amnesic multi-domain cognitive impairment.

No Cerebrospinal fluid (CSF) biomarkers were available for all the patients (LOAD, VD, and CIND) enrolled into this study.

Forty cognitively normal older individuals were enrolled as controls (C). Their mean age was 72 ± 8.7 SD years; females represented 68% of the sample. All these individuals were free-living, healthy (no important comorbidity was found), and were independent on ADLs (mean Barthel score: 96/100). Their average MMSE score was 28/30. In case of memory complains, a battery of tests was administered, as previously described [20].

For the evaluation of the PPARgamma Pro12Ala polymorphisms, additional 104 normal older individuals were enrolled (mean age: 77 ± 9.1 SD years; females: 52.6%).

Personal data and medical history were collected by a structured interview from patients and caregivers. All patients underwent a general and neurological examination; clinical-chemistry analyses were performed to exclude causes of secondary cognitive impairment. The diagnosis of dementia was made by trained geriatricians; for neuropsychological assessment, all patients were given a battery of tests [20]. Subjects affected by severe congestive heart failure (New York Heart Association class III-IV), severe liver or kidney disease, severe chronic obstructive pulmonary disease, and cancer were excluded. There were no evidences of acute illnesses at the time of clinical observation in DH and blood sampling.

The criteria for the diagnosis of diabetes were: 1) history of diabetes or current hypoglycaemic therapy; 2) fasting glycaemia > 126 mg/dl in two or more measurements; 3) glycaemia > 200 mg/dl at 120 min after oral glucose load. The criteria for the diagnosis of hypertension were: 1) history of hypertension or antihypertensive therapy at visit time; 2) blood pressure > 140/90 mmHg in three or more measurements. The diagnosis of coronary heart disease (CHD) was made in the presence of a documented history of previous myocardial infarction or angina. No patients were taking a statin at the time of enrolment into the study. All subjects (and/or their caregiver if demented) were informed about the research project during the first visit, and gave their written consent in order to participate to the study. The study was approved by the local ethic committee (S. Anna University Hospital, Ferrara, Italy) and was conducted in accordance with the Helsinki Declaration as revised in 1989.

### Brain CT scan

All patients (LOAD, VD, and CIND) underwent a brain CT. The instrument used was a third generation SIEMENS SOMATON HQ. The slice thickness was 10 mm. Radiograms were evaluated by trained radiologists not informed about the clinical characteristics of the patient. CT scan information was used to support the clinical diagnosis, and to exclude possible brain pathologies associated with cognitive impairment. CT scan was also used for a qualitative evaluation of brain morphology. Brain atrophy was assessed through direct/indirect signs of neuronal loss. Lacunar infarcts were defined as demarcated lesions with a diameter < 15 mm involving deep regions such as internal capsulae, basal ganglia, corona radiata or thalamus. Cortical infarctions were defined as the presence of large cortical-subcortical infarctions resulting from the occlusion of large cerebral arteries.

### Plasma 24S-OH-Chol concentrations measurement

Blood was drown in the morning after a 12-hours overnight fasting, and after the patient has been sedentary in sitting or supine position for at least 10 minutes. After having been aliquoted, plasma was frozen and stored at -80°C until the tests were performed.

Stock solutions of 24S-OH-Chol and racemic 24OHChol-d_6 _internal standard (IS), obtained from Avanti Polar Lipids, were prepared in toluene at a concentration of 1 mg/mL. Samples were purified following a procedure previously described by DeBarber et al. [21], after adding 5 μL of 5 μg/mL isopropanol IS working solution, to 500 μL of serum. The dried organic extract residue was reconstituted in 125 μL of acetonitrile/methanol (30/70 v/v). 24S-OH-Chol concentration was determined using a linear 7-point calibration curve ranging from 7.85 to 500 ng/mL, prepared evaporating an aliquot of the 24S-OH-Chol working isopropanol solutions under flow of pure N_2 _and redissolving the dried sample with an appropriate volume of acetonitrile/methanol (30/70 v/v). 24S-OH-Chol assay was performed using an ultra performance liquid cromatograph (Dionex Ultimate 3000 RS) coupled with a mass spectrometer (AB SCIEX Triple Quadrupole API 3000), equipped with an atmospheric pressure chemical ionization (APCI) source and operating in multiple reaction monitoring in positive mode. Transition from *m/z *385.4 to *m/z *367.5 ion was monitored for 24S-OH-Chol and transition from *m/z *391.4 to *m/z *373.5 ion for 24OHChol-d_6. _Chromatographic separation was performed using a Pinnacle DB C18 column (1, 9 um particle size; 100 × 2.1 mm i.d.; Restek) at a temperature of 50°C, with a mobile phase consisted of MeOH/CH_3_CN/H_2_O 45/45/10+ 0.1% acetic acid (phase A) and CH_2_Cl/MeOH (80/20 v/v) (phase B). The eluition gradient was programmed as follows (flow rate, 0.8 mL/min): 100% mobile phase A for 5 minute, increased to 100% B in 2 minutes, 5 minutes at 100% B, decreased to 100% A in 1 minute and then reequilibrated with 100% A for 10 minute until the next sample was injected. The linear regression constant (r) was more than 0.999 in the linear range 7.85-500 mg/mL. Calibrators accuracy and interday precision (CV%) ranged respectively from 95.81%-108.5% and 0.6%-11.96%. Recovery was between 102,68% and 95,92%. Intra- and interday precision were respectively less than 5% and 8% for calculated 24S-OH-Chol. The detection limit was 1 ng/mL and the quantification limit 5 ng/mL. 24S-OH-Chol was reported as plasma levels (ng/ml).

### Plasma cholesterol measurement

Plasma concentrations of total cholesterol (TC) were measured by standard enzymatic procedures (CHOD-PAP Method, Boehringer, Mannheim, Germany).

### PPARgamma Pro12Ala genotyping

Genomic DNA was extracted from blood leukocytes by the salting out method. The Pro12Ala PPARgamma variant was detected by polymerase chain reaction-restriction fragment length polymorphism (PCR-RFLP) analysis. This analysis a mutagenic PCR primer is used to introduce a BstUI restriction site only when a C→G substitution at nucleotide 34 of the PPARgamma 2 gene is present [22]. Genotyping was repeated for all Ala12 homozygotes and Pro12Ala heterozygotes; reproducibility was 100%.

### Statistical analysis

Continuous variables were expressed as mean (SD) or median (interquartile range) when necessary. The distribution of 24S-OH-Cholesterol values was skewed; consequently, data were expressed as median values (interquartile range). Means were compared by ANOVA using the Fisher's least significant difference (LSD) test for post-hoc analysis. 24S-OH-Cholesterol values were LOG-tranformed before entering ANOVA. Bivariate correlations were tested by the Pearson's test. Prevalence was compared by the χ^2 ^test. Multivariate linear regression analysis (method stepwise forward) was used to test the association between the 24S-OH-Chol/TC ratio and other variables previously selected by univariate analysis. Dicotomous variables were included as dummy variables (0: absent; 1: present).

SPSS for Windows, version 7.0 (SPSS, Inc, Chicago, IL)statistical packages were used.

## Results

In Table 1 are reported the general characteristics and the plasma levels of 24S-OH-Chol in patients with LOAD, VD, CIND, and in C. The prevalence of female gender was higher in LOAD and lower in VD compared with the other groups. Mean age was lower, while MMSE score was higher in C compared with the other groups. The Barthel index score was higher in C and CIND compared with LOAD and VD. Brain atrophy on CT scan was more frequently reported in LOAD patients, while ischemic lesions (both lacunar and cortical infarcts) were more frequent in VD (Chi square: all p: 0.001).

**Table 1:** General characteristics and plasma levels of 24S-OH-Chol in patients with Late Onset Alzheimer's Disease (LOAD), Vascular Dementia (VD), Cognitive Impairment No Dementia (CIND), and in older controls.

Parameter	LOAD	VD	CIND	Controls
	(n. 60)	(n. 35)	(n. 25)	(n. 40)
Age (years)*	78 ± 5.5	77 ± 7.6	74 ± 8.2	73 ± 8.7
**Female gender ^**	78,00%	53,00%	60,00%	68,00%
MMSE score (mean/SD)*	21.3 ± 3.8	21.7 ± 3.9	25 ± 3.1	28 ± 2
**Barthel index **§	82.5/100	79/100	96/100	96/100
**GDS**	5.7/15	5.3/15	6.3/15	6.2/15
**Tot. Chol. (mg/dL)**	210 ± 42	204 ± 32	207 ± 43	215 ± 28
**Albumin (g/dL)**	4.14 ± 0.38	4.11 ± 0.37	4.08 ± 0.34	4.22 ± 0.31
**Hypertension**	67,00%	83,00%	63,00%	67,00%
**Diabetes**	15,00%	34.5%	18,00%	19,00%
**CHD ^**	16,00%	34.5%	12,00%	9.5%
**24S-OH-Chol (ng/ml)°**	51.0 (42.0-64.7)	39.1 (36.0-46.5)	47.0 (42.6-56.3)	46.3 (37.5-52.7)
**Brain atrophy **^^	84%	53%	51%	47%
**Single lacune **^^	5%	22%	7%	6.5%
**Multiple lacunes **^^	0%	55%	20%	10%
**Cortical infarcts **^^	0%	23%	11%	6%

* ANOVA (model P < 0.001) Fisher's least significant difference (LSD) post-hoc test: C vs LOAD p < 0.01; C vs VD p < 0.05; VD vs LOAD and CIND p < 0.005

§ ANOVA (model P < 0.001) LSD p < 0.05 C and CIND vs LOAD and VD

^Chi square test p < 0.05; ^^Chi square test p < 0.001

° ANOVA (model P < 0.001) LSD p < 0.01 LOAD vs C; p < 0.05 VD vs C and CIND; p < 0.01 for LOAD vs VD

MMSE: mini mental state examination; GDS: geriatric depression scale; CHD: coronary heart disease. For 24S-OH-Chol: median (interquartile range)

Compared with C, plasma 24S-OH-Chol levels were higher in LOAD (LSD post-hoc test p: 0.01) and lower in VD (LSD p: 0.05), while no differences were observed as regards the CIND group (model ANOVA p < 0.001).

**Figure 1:**
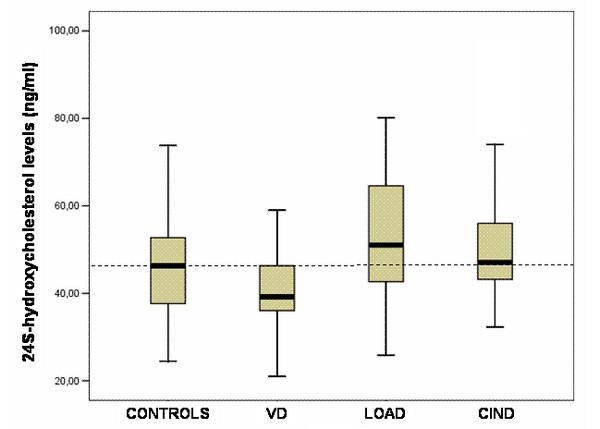
**24 Shydroxycholesterol plasma levels (boxplots) in older normal individuals (Controls) and in older subjects affected by vascular dementia (VD), late onset Alzheimer's disease (LOAD) or cognitive impairment-no dementia (CIND)**. The dashed line represents the median value of plasma 24Shydroxycholesterol in controls (46.3 ng/ml).

The distribution of 24S-OH-Chol levels (boxplots) in Controls and patients affected by VD, LOAD or CIND is reported in Figure 1.

In Table 2 are reported the correlations between the 24S-OH-Chol and other variables observed in the whole sample (n. 160 subjects). Since 24S-OH-Chol and TC plasma levels are known to be correlated [4] (in our sample r: 0.28, p: 0.005) as they are both transported by the low-density lipoproteins, 24S-OH-Chol values were adjusted for TC levels by calculating the 24S-OH-Chol/TC ratio (ng/mg). Indeed, it has been suggested that the 24S-OH-Chol/TC ratio may better reflect brain cholesterol homeostasis than 24S-OH-Chol absolute level [4].

**Table 2:** Pearson's correlations between the 24S-OH-Chol/TC ratio and other variables in the whole sample (160 individuals).

Variable	R	P
**Age**	0.11	0.18
**Barthel index**	-0.06	0.70
**Serum Albumin**	-0.20	**0.03**
**Serum Creatinine**	0.04	0.96
**Hs.CRP**	0.33	**0.001**
**MMSE**	-0.07	0.69
**Rey test (short)**	-0.14	0.17
**Rey test (long)**	-0.15	0.14
**Token test**	-0.06	0.71
**Verbal fluency (letter)**	-0.02	0.79
**Verbal fluency (category)**	-0.11	0.27
**Babcock (immediate)**	-0.29	**0.01**
**Babcock (delayed)**	-0.22	**0.03**
**FAB**	0.26	**0.04**
**Trail making A**	0.18	0.31
**Trail making B**	0.03	0.89
**CT SCAN IMAGING**		
**- Atrophy**	0.17	**0.05**
**- Cortical infarction**	-0.04	0.70
**- Single lacune**	0.01	0.84
**- Multiple lacunes**	-0.16	0.06

MMSE: mini mental state examination; FAB: frontal assessment battery

The 24S-OH-Chol/TC ratio was significantly correlated with serum albumin (r: -020; p: 0.03), and hs.CPR levels (r: 0.33; p: 0.001); no significant correlations emerged with age, Barthel index, and creatinine levels. Interestingly, the 24S-OH-Chol/TC ratio correlated negatively with the Babcock test score (both immediate and delayed recall), and positively with the Frontal Assessment Battery (FAB) score. In both cases, the higher the 24S-OH-Chol/TC ratio, the worse the performance obtained in neuropsychological tests.

As regards TC scan findings, 24S-OH-Chol/TC ratio was positively related to the presence of brain atrophy. By multivariate linear regression analysis we demonstrated that the 24S-OH-Chol/TC ratio was significantly correlated with hs.CRP independent of age, albumin levels, brain atrophy, Babcock test, and FAB score (ANOVA: p:0.04; r^2^: 0.104).

In Table 3 are reported the results of PPARgamma Pro12Ala polymorphism genotyping in patients with LOAD, VD, or CIND, and in 144 older controls. The distribution of the two alleles was in Hardy-Weinberg equilibrium. No differences emerged by comparing their distribution in the four groups of subjects. Furthermore, no differences in median/mean plasma 24S-OH-Chol levels were found when individuals bearing the Ala allele (heterozygous + homozygous) were compared with Pro/Pro homozygote subjects (data not shown).

**Table 3:** PPARgamma Pro12Ala polymorphism in older patients with LOAD, VD, CIND, and in older controls.

	LOAD	VD	CIND	Controls
	(n. 60)	(n. 35)	(n. 25)	(n. 144)
**Pro allele**	0.92	0.91	0.89	0.92
**Ala allele**	0.08	0.09	0.11	0.08
				
**Homo Pro/Pro**	48	29	19	126
**Etero Pro/Ala**	10	6	6	16
**Homo Ala/Ala**	2	0	0	2

## Discussion

We evaluated the plasma levels of 24S-OH-Chol in a sample of aging patients affected by two different forms of dementia, LOAD and VD, and in individuals with CIND. The first interesting data emerging from our study is the finding of higher circulating levels of 24S-OH-Chol in patients with LOAD compared with Controls. Our results seem to confirm the unique observation by Lutjohann et al. [12], while are in contrast with following studies reporting decreased levels of 24S-OH-Chol in LOAD patients [13-15]. Unlike that study [12], we were not able to demonstrate in LOAD patients a significant correlation between 24S-OH-Chol levels and the global cognitive function (MMSE) or functional impairment (Barthel index), perhaps because our subjects were not in the very-early stage of the disease (Global Deterioration Scale: stage 3 to 5). Our data support the hypothesis of a trend toward higher levels of plasma 24S-OH-Chol in the early stages of LOAD, when the rate of neurodegeneration is higher than normal, but the amount of cell loss and resulting brain atrophy is still small. The precise mechanisms leading to increased plasma 24S-OH-Chol levels in the early stages of LOAD are not known. It is possible that, in these specific conditions, cholesterol turnover might be increased in CNS (due to neuronal degradation) and/or that the rate of conversion of brain cholesterol to 24S-OH-Chol might be higher compared to normal.

Interestingly, it has been observed by Brown et al. that 24S-OH-Chol is an efficient inhibitor of beta-amyloid formation in vitro [23]; if this is true also under in vivo conditions, the increase of 24S-OH-Chol levels in CNS (and consequently in the circulation) might be considered as an early attempt to counteract beta-amyloid deposition. On the contrary, in the advanced stages of the disease low levels of 24S-OH-Chol in CNS (and consequently in the circulation), would even accelerate beta-amyloid deposition and the progression of LOAD.

In part, increased 24S-OH-Chol levels might also result from a defect in blood-brain barrier, which seems to be a frequent finding in different neurological diseases including LOAD [24].

Unlike the study of Lutjohann et al. [12], but in agreement with other following clinical observations [13-15], we found that plasma 24S-OH-Chol levels were lower in VD patients compared with controls. The difference we observed between LOAD and VD is somehow unexpected, and might be related to different mechanisms. In particular, it has to be underlined that, on average, our VD patients were in a more advanced stage of the disease (GDS: stage 4-6) compared with LOAD (GDS: stage 3-5). Actually, not only it has been reported that a decrease in plasma levels of 24S-OH-Chol would be typical of dementia [25,8], but it has been also shown that plasma 24S-OH-Chol levels progressively decrease with the worsening of the disease [4]. Thus, the differences between LOAD and VD might be secondary to a different stage of disease. Alternatively, the differences in levels of 24S-OH-Chol between LOAD and VD might reflect the different pathogenetic mechanisms and/or evolution of these two forms of dementia.

We also evaluated the possible relationship between 24S-OH-Chol and other available variables. Interestingly, by multivariate regression analysis we found that the 24S-OH-Chol/TC ratio independently correlated with hs.CRP levels, and this explained about 10% of its total variability. Hs.CRP is a sensitive marker of systemic inflammation. The association between the 24S-OH-Chol and inflammation has been already reported *in vitro *[26]; indeed, Alexandrov found that in primary co-culture of human neurons and glia, 24S-OH-Chol is able to induce the expression of several pro-inflammatory genes. The presence of a low-grade systemic inflammation has been reported both in LOAD and VD by several Authors, including our group [20]. The finding of a significant association between 24S-OH-Chol and CRP levels suggest a possible link between the degree of neurodegeneration (plasma 24S-OH-Chol would be a marker of it) and the degree of peripheral systemic inflammation. As a matter of fact, the relationship between 24S-OH-Chol/TC and CRP was strong in LOAD (r: 0.39), was still present in CIND (r: 0.20), but it was pratically absent in VD patients (r: 0.08). The specificity of the relationship between LOAD and increased 24S-OH-Chol plasma concentrations might be indirectly supported by the result of univariate analysis (Table 2) showing a significant correlation of 24S-OH-Chol with memory tests impairment and brain atrophy on CT scan, both typical characteristics of LOAD but not of VD.

Finally, we evaluated the possible effect of the PPARgamma Pro12Ala polymorphism on the risk of being affected by dementia or CIND, as well as on 24S-OH-Chol plasma levels. Infact, it has been consistently reported that PPARgamma plays an important role in glucose and lipid metabolism [27], which in turn have been associated with LOAD [28,29]. We found no association between the PPARgamma polymorphism and LOAD, VD or CIND. Furthermore, unlike Sauder [17] we didn't find any increase in 24S-OH-Chol/TC ratio among the carriers of the Ala allele both in the whole sample and in the three groups of patients.

The principal limitations of the study must be also acknowledged. First, brain morphology was evaluated by qualitative CT scan assessment and not by quantitative MRI analysis, which is the best validated method and would give important information. Second, CSF biomarkers were not available for LOAD, VD and CIND patients enrolled into this study, and this might significantly influence sensibility and specificity of our diagnoses. Third, we did not systematically evaluated Apo E polymorphism in our sample, and it is probable that apo E phenotype might influence plasma levels of 24S-OH-Chol. Nevertheless, we evaluated apo E genotype in DNA from 70% of patients (84/120); we found no differences in mean/median 24S-OH-Chol levels by comparing patients bearing or not the ε4 allele (ANOVA - Mann-Whitney test p: 0.86 and 0.96, respectively).

Fourth, although we investigated plasma 24S-OH-Chol concentrations in a much larger sample of subjects compared with previous studies, sample size might be small when investigating the possible effect of genetics. For this reason our negative data on PPARgamma polymorphism need to be replicated in larger groups of individuals.

## Conclusions

In conclusion, the results of the present study suggest that 24S-OH-Chol plasma levels might be increased in the first stages of LOAD, and may be correlated with systemic inflammation.

The finding of lower 24S-OH-Chol concentrations in VD might be related with a more advanced stage of VD compared with LOAD in our sample, and/or to different pathogenetic mechanisms and evolution of these two forms of dementia.

## Competing interests

The authors declare that they have no competing interests.

## Authors' contributions

GZ contributed to conception, design, and acquisition of data and manuscript drafting; MPD participated to analysis and interpretation of data and revised the manuscript critically; CB contributed to acquisition and interpretation of data and revised the manuscript critically; AP participated to analysis and interpretation of data and manuscript drafting; EDN contributed to conception and interpretation of data and revised the manuscript critically; AZ participated to conception and interpretation of data, and revised the manuscript critically; FB contributed to conception of data and revised the manuscript critically; AFM participated to analysis and interpretation of data and revised the manuscript critically; CC contributed to conception, analysis, and interpretation of data and revised the manuscript critically. All the Authors gave the final approval to the version to be published.

## Pre-publication history

The pre-publication history for this paper can be accessed here:

http://www.biomedcentral.com/1471-2377/11/121/prepub
